# *Cysticercus tenuicollis* in selected locations in Poland: genetic diversity, prevalence and epidemiological patterns in roe deer *(Capreolus capreolus)* and moose *(Alces alces)*

**DOI:** 10.2478/jvetres-2026-0007

**Published:** 2026-02-12

**Authors:** Anna Maria Pyziel, Joanna Banasiewicz, Katarzyna Filip-Hutsch, Kateryna Slivinska, Marta Kloch, Marcin Świątek, Olena Zhytova, Michalina Gmaj, Daniel Klich

**Affiliations:** Department of Public Health Protection and Animal Welfare, Faculty of Biological and Veterinary Sciences, Institute of Veterinary Medicine, Nicolaus Copernicus University in Toruń, 87-100 Toruń, Poland; Department of Biochemistry and Microbiology, Institute of Biology, Department of Food Hygiene and Public Health Protection, Institute of Veterinary Medicine, Warsaw University of Life Sciences, 02-776 Warsaw, Poland; Department of Food Hygiene and Public Health Protection, Institute of Veterinary Medicine, Warsaw University of Life Sciences, 02-776 Warsaw, Poland; Museum and Institute of Zoology of the Polish Academy of Sciences, 00-818 Warsaw, Poland; I.I. Schmalhausen Institute of Zoology of the National Academy of Sciences of Ukraine, 01030 Kyiv, Ukraine; Department of Animal Genetics and Conservation, Department of Animal Breeding and Nutrition, Institute of Animal Sciences, Warsaw University of Life Sciences, 02-787 Warsaw, Poland; Department of Animal Breeding and Nutrition, Institute of Animal Sciences, Warsaw University of Life Sciences, 02-787 Warsaw, Poland; Polissia National University, 10008 Zhytomyr, Ukraine

**Keywords:** *cox*1, habitat fragmentation, metacestode, *Taenia hydatigena*, wild ruminant ungulates

## Abstract

**Introduction:**

*Taenia hydatigena* is a widespread tapeworm. The predilection site of the adult form is the small intestine of its carnivore definitive hosts, and this site of the larval form, *Cysticercus tenuicollis*, is the abdominal visceral organs of its ungulate intermediate hosts. Cysticercal lesions are of food safety and economic importance, as they may condemn carcasses or internal organs of slaughtered animals and hunted game. The study aimed to evaluate the prevalence, intensity of infection and species composition of metacestodes from Polish roe deer and moose, and analyse the genetic diversity of the isolates.

**Material and Methods:**

Altogether, 167 roe deer (from the Mazowieckie and Łódzkie voivodeships) and 36 moose (from the Mazowieckie, Lubelskie and Podlaskie voivodeships) were studied. Metacestodes were collected post mortem and used for molecular investigations based on the partial *cox*1 (cytochrome *c* oxidase subunit 1) gene.

**Results:**

The prevalence and the general intensity of infection were 9.6% and 1–6 cysts for roe deer, and 8.3% and 1–9 cysts for moose. Exclusively *T. hydatigena* infections were noted. Intraspecific genetic diversity of 1.42% was observed. The omentum and the mesentery were the most prevalent locations of metacestodes.

**Conclusion:**

*Taenia hydatigena* isolates from roe deer and moose varied genetically. Assuming national prevalence at the level observed in this study, the number of infected roe deer in Poland could exceed 18,000. Although *T. hydatigena* is not a zoonotic agent, its spread should be monitored and limited. Dogs should not be fed raw game meat in order to prevent parasitosis from spreading to farm animals.

## Introduction

*Taenia hydatigena* Pallas, 1766 (Cestoda, Cyclophillidea), is a widespread tapeworm with an indirect life cycle (11, 25). The adult forms of the parasite are found in the small intestine of domestic and wild carnivores such as dogs, cats, wolves, foxes, jackals, raccoons, lynx and bears, which are the definitive hosts (20). The larval form of the parasite, *Cysticercus tenuicollis*, can be found in the abdominal visceral organs of ungulates, including cattle, sheep, goats, horses, pigs, wild boar and deer, which are the intermediate hosts (3, 21, 23). The presence of cysticerci is usually exposed during the official inspection of a carcass and internal organs of slaughtered animals and hunted game (13). *Taenia hydatigena* metacestodes are generally observed on the visceral surface of the liver and in the omentum, peritoneum and mesentery, and, less often, occur on the pleura and pericardium and in the lungs, kidneys, brain, ovaries, uterus, and even the uterine tubes of intermediate hosts (1, 6, 9, 11). An intermediate host becomes infected by ingesting feed and water contaminated with the tapeworm’s eggs, which are excreted in gravid proglottids in the definitive host’s faeces. The larvae hatch in the small intestine, and subsequently migrate to their final location. The infection can be dangerous for young animals, as the migrating larvae of *T. hydatigena* can cause traumatic hepatitis (2, 22). The life cycle of the parasite is complete when a definitive host preys on the infected ungulate. The species identification of cysticerci can be challenging for meat inspectors, as the metacestodes of *T. hydatigena* may be mistaken for those of *T. lynciscapreoli* (10). Although the life cycle, location and appearance of the cysticerci of both tapeworms are alike, *T. lynciscapreoli* prefers the Eurasian lynx as its main definitive host, and the roe deer as its main intermediate host (10). Additionally, the species was also found in the wolf, as well as in the moose and reindeer, as definitive and intermediate hosts, respectively (10, 13). Both *T. hydatigena* and *T. lynciscapreoli* have been reported in Poland previously. *Taenia hydatigena* has long been found in cervids, wild boar, moose and pigs in Poland (4, 5, 6, 7, 14, 37). In contrast, the history of reporting *T. lynciscapreoli* in Poland is quite recent, as the species was noted for the first time in 2018, when it was found in a lynx (17). Further investigation confirmed infections in lynx and roe deer in Poland (24). It is worth mentioning that neither of the species is a zoonotic agent, in contrast to *T. solium* and *T. saginata* (8). This research concentrated on the roe deer and moose as intermediate hosts for *T. hydatigena* and *T. lynciscapreoli*, and evaluated the prevalence, intensity of infection and species composition of metacestodes found in both host species. The genetic diversity of Polish isolates of *Taenia* sp. was analysed based on the cytochrome *c* oxidase subunit 1 (*cox*1) genetic marker.

## Material and Methods

### Examined animals and the area of the study

**Fig. 1.**
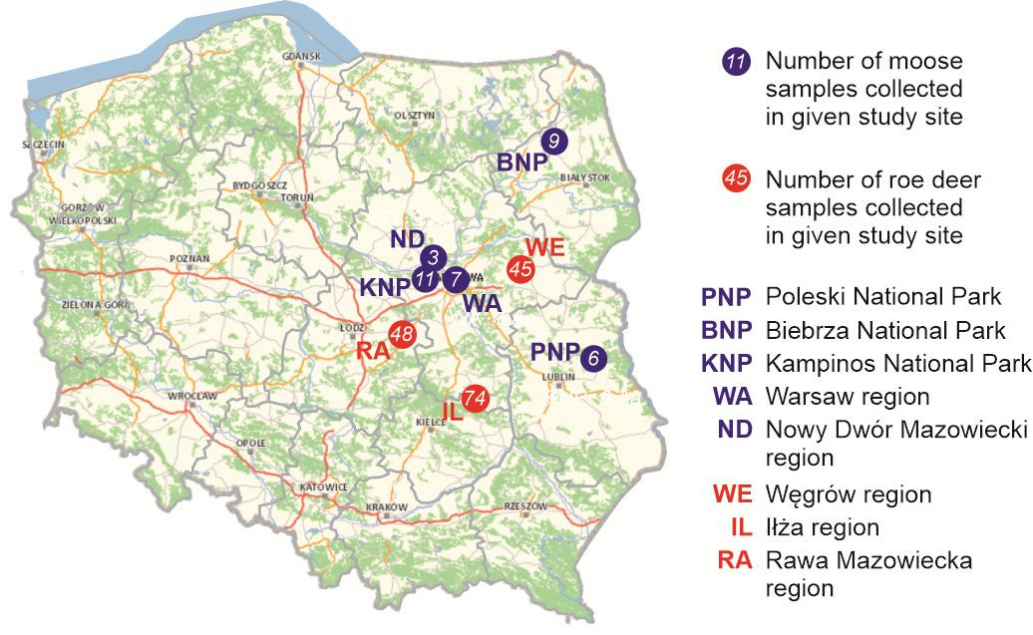
Location of collection sites of roe deer and moose samples in Poland

Altogether, 167 roe deer and 36 moose were included in the study. The samples from roe deer were obtained during 2022–2023 and 2023–2024 seasons from animals taken as hunt prey compliantly with the Polish Hunting Act. Research was conducted on material from 45 roe deer from the Węgrów region (52°22′34″N, 21°47′39″E) and 74 from the Iłża region (51°11′5″N, 21°9′45″E), both in the Mazowieckie voivodeship in central Poland, and on material from 48 roe deer from the Rawa Mazowiecka region (51°48′25″N, 20°7′35″E) in the Łódzkie voivodeship also in central Poland (Fig. 1). All roe deer included in the study lived in and around large wind farms.

Materials from roe deer were collected during field dissection by hunters with standard techniques just after the animal’s death, using the Polish protocol for animal infectious diseases. Materials from moose were also collected during field dissection with standard techniques, no more than two days after the animal’s death, but by a qualified veterinarian. The sex of the animal was also determined, and its age was estimated by hunters on the basis of tooth wear. After all collected material had been transported to the laboratory, it was examined by veterinarians and veterinary parasitologists for the presence of metacestodes. The samples were placed in sterile containers and preserved in 70% ethanol for further molecular analyses.

Moose samples were collected in the years 2017–2025 from animals killed by vehicle strikes or found dead. The study was conducted on 21 moose from the Mazowieckie voivodeship in central Poland, these being 11 animals from the Kampinos National Park (52°19′12″N, 20°38′38″E), 7 individuals from the Warsaw region (52°14′0.9″N, 20°58′56″E) and 3 animals from the Nowy Dwór Mazowiecki region (52°28′17″N, 20°42′49″E). Additionally, 9 individuals were found in the Biebrza National Park (53°2945N, 22°457E) in the Podlaskie voivodeship, north-eastern Poland, and 6 animals came to light in the Polesie National Park (51°25′53″N, 23°7′7″E) in the Lubelskie voivodeship in eastern Poland. The age of the moose was determined based on tooth wear by experienced field personnel, *i.e*. forest and park rangers and hunters (31). The age of some moose from national parks, which are subjected to long-term monitoring, could be verified through observation and individual records.

### Extraction, amplification and sequencing of DNA

Genomic DNA was extracted individually from ethanol-preserved metacestodes (35 from roe deer and 13 from moose) using a NucleoSpin Tissue DNA extraction kit (Macherey-Nagel, Düren, Germany) according to the manufacturer’s protocol. A partial region of the mitochondrial *cox*1 gene was amplified using the following set of primers: Thg452F (5′-TGCATTTAGCTGGTGCGTCAAGTA-3′) forward and Thg1326R (5′-ACAAACACGCCGGGGTAACC-3′) reverse (6). A polymerase chain reaction was performed in a T100 thermal cycler (Bio-Rad, Hercules, CA, USA) in a volume of 50 μL. Each 50 μL PCR reaction contained 20 μL of Molecular Biology Reagent Water (Sigma-Aldrich, St. Louis, MO, USA), 25 μL of AccuStart II PCR ToughMix (×2 concentration) (Quantabio, Beverly, MA, USA), 1 μL of GelTrack Loading Dye (×50 concentration) (Quantabio), 1 μL of forward primer (20 mM), 1 μL of reverse primer (20 mM) and 2 μL of template DNA. The conditions for PCR were as follows: 94°C for 2 min to denature the DNA; 35 cycles at 94°C for 40 s, 56°C for 40 s and 72°C for 40 s; and a final extension of 5 min at 72°C to ensure complete amplification. The PCR product was purified with the use of the NucleoSpin Gel and PCR Clean-up kit (Macherey-Nagel), eluted with 30 μL of Molecular Biology Reagent Water (Sigma-Aldrich), and sequenced in both directions by Genomed S.A. (Warsaw, Poland) using the primers used for amplification (5 mM). The sequences were then assembled into contigs using CodonCode Aligner v. 8.0 (CodonCode, Centerville, MA, USA). The obtained nucleotide sequences were compared to the NCBI database of sequences using BLAST.

### Phylogenetic analysis

Phylogenetic analysis was performed based on the described sequenced DNA fragment of the *cox*1 marker gene using 48 sequences of *Taenia hydatigena*, with *T. lynciscapreoli* (GenBank accession No. MK033479) as an outgroup (Table 1). The sequences were aligned using ClustalW software (18), and the alignment was trimmed to the length of the shortest sequence (776 bp). Maximum-likelihood phylogenies were inferred with MEGA 6 (36) using the best-fit nucleotide substitution model (*i.e*. General Time Reversible, GTR+I (invariant sites) +G (Γ distribution) as indicated by jModelTest v. 2.1.4 (30). Branch support was estimated using nonparametric bootstrap analyses based on 1,000 replicates.

**Table 1.** List of taxa included in the molecular analysis using *cox*1 sequence data

Species	Host	Country	Region	GenBank accession Nos
*T. hydatigena*	*Capreolus capreolus*	Poland	Węgrów	OR711631–OR711633, PP387596, PP387598–PP387600, PP387611, PP387612
*T. hydatigena*	*Capreolus capreolus*	Poland	Iłża	OR711638, OR830597–OR830599, PP387620, PQ157678–PQ157680, PQ157682–PQ157685, PQ525696, PQ525700
*T. hydatigena*	*Capreolus capreolus*	Poland	Rawa	OR711634, OR711635, OR711637, OR830596, PP387619
*T. hydatigena*	*Capreolus capreolus*	Poland	Gorlice	PP408282
*T. hydatigena*	*Alces alces*	Poland	Biebrza NP	PP408282–PP408290
*T. hydatigena*	*Alces alces*	Poland	Polesie NP	PP408295
*T. hydatigena*	*Alces alces*	Poland	Kampinos NP	PP408291–PP408293, MF630924, MF630925
*T. hydatigena*	*Canis familiaris*	China	No data	MT784872
*T. hydatigena*	*Ovis aries*	Ghana	No data	MK945749
*T. hydatigena*	*Ovis aries*	Turkey	No data	OQ317803
*T. hydatigena*	*Capra hircus*	Turkey	No data	OQ317833
*T. lynciscapreoli*	*Lynx lynx*	Poland	Bieszczady	MK033479

1 NP – National Park

### Statistical analysis of *Cysticercus tenuicollis* presence in roe deer

A large number of roe deer samples were available, lending themselves to a simple statistical analysis of the dependence of the prevalence of metacestodes on the sex of animals, the study area and the presence of animals in the wind farm area. In the analysis of these samples, three study areas were included, chosen because they were 100 km or more apart: Węgrów, Iłża and Rawa Mazowiecka. The existence of any correlation between prevalence of metacestodes in roe deer and animal proximity to wind farms was assessed to take account of a possible impact of this infrastructure on the health and condition of wild and farm animals (12, 15, 16, 20). The analysis was performed with the chi-squared test of independence in IBM SPSS Statistics 29.0 (Armonk, NY, USA).

## Results

### Prevalence and intensity of infection

**Fig. 2.**
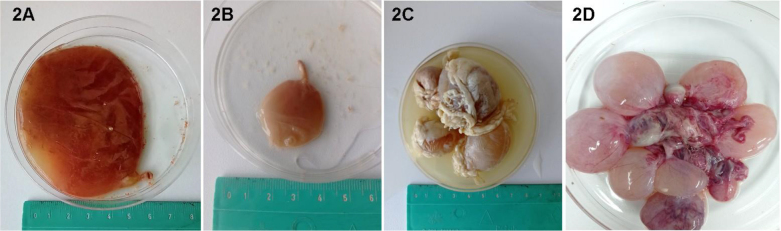
Metacestodes found in roe deer and moose. 2A: metacestode isolated from the omentum of a roe deer from the Węgrów region; 2B: metacestode isolated from the liver of a roe deer from the Rawa Mazowiecka region; 2C: metacestodes isolated from the omentum of a roe deer from the Iłża region; 2D: metacestodes isolated from the mesentery of a moose from the Biebrza National Park

Single or cluster-like metacestodes approximately 2–8 cm in diameter were observed in 16 of 167 dissected roe deer and 3 of 36 examined moose (9.6% prevalence for roe deer and 8.3% for moose) (Fig. 2). The general intensity of infection ranged from one to six in roe deer (Tables 2 and 3), and one to nine in moose (Table 4). Metacestodes were the most prevalent in roe deer from the Iłża region, as they were diagnosed in 9 of 74 animals (12.2% prevalence), and their prevalence was similar in individuals from the Rawa Mazowiecka and Węgrów regions (8.2% and 6.7% respective prevalence). The highest intensity of infection in roe deer, which was six cysts, was found in one individual from the Węgrów region. The maximal number of cysts was four in animals from the Iłża and Rawa Mazowiecka regions. The highest intensity of infection in moose, reaching nine cysts, was noted in an animal from the Biebrza National Park; three cysts were found in the moose from Kampinos National Park and a single cyst in the individual from Polesie National Park. The prevalence of metacestodes in roe deer did not statistically depend on sex (χ^2^ = 0.18, P-value = 0.670), study area (χ^2^ = 1.10, P-value = 0.578) or proximity to a wind farm (χ^2^ = 0.33, P-value = 0.564).

**Table 2.** Metacestodes found in roe deer in the Iłża region

Animal			Metacestode	
No.	Sex	Age (yrs)	Location	GenBank accession Nos
1	F	3	Omentum	OR711638, OR711639
2	F	3	Omentum	OR830597, OR830598
3	M	4	Omentum	OR830599
4	F	5	Omentum	PP387620
5	F	4	Omentum	PQ157678–PQ157680
6	F	3	Omentum	PQ157682
7	F	4	Anus	PQ157683, PQ157684
			Omentum	PQ157685
8	M	6	Omentum	PQ525696–PQ525699
9	M	7	Omentum	PQ525700

**Table 3.** Metacestodes found in roe deer in the Rawa Mazowiecka and Węgrów regions

Animal					Metacestode
No.	Region	Sex	Age (yrs)	Location	GenBank accession Nos
1	RA	F	4	Liver	OR711634–OR711636
				Omentum	OR711637
2	RA	M	4	Omentum	OR830596
3	RA	F	4	Omentum	PP387619
4	RA	F	4	Bladder	PQ157681
1	WE	F	3	Mesentery	OR711631–OR711633
2	WE	M	8	Omentum	PP387596
3	WE	M	4	Omentum	PP387597–PP387600
				Omentum	PP387611, PP387612

1 RA – Rawa Mazowiecka; WE – Węgrów

**Table 4.** Metacestodes found in moose inhabiting Biebrza, Kampinos and Polesie national parks

No.	Region	Sex	Age (yrs)	Location	GenBank accession Nos
1	BNP	M	4	Mesentery	PP408282–PP408290
1	KNP	M	3	Mesentery	PP408291–PP408293
1	PNP	M	<1	Liver	PP408295

1 BNP – Biebrza National Park; KNP – Kampinos National Park; PNP – Polesie National Park

### Cyst location

The omentum was the most prevalent location for cysticerci in roe deer. A total of 26 tapeworm cysts were found in the omenta of 14 animals from all three examined locations. Less prevalent locations were the mesentery, with three cysts found in one individual from the Węgrów region; the anus, with two cysts found in one roe deer from the Iłża region; and the bladder, where one cyst was located in one individual from the Rawa Mazowiecka region. The mesentery was the most prevalent location of the metacestodes in moose, as 12 cysts were found together in two individuals: one from the Biebrza National Park and the second from the Kampinos National Park. In one moose from Polesie National Park, one cysticercus was noted on the liver capsule.

### Nucleotide sequences

The study yielded 48 novel DNA sequences of the partial *cox*1 gene from cysticerci of roe deer and moose. All of them corresponded to *Taenia hydatigena* and were from 98.58% to 100% homologous (Supplementary Table 1). Those were 35 sequences derived from roe deer metacestodes (Tables 2 and 3) and 13 derived from moose metacestodes (Table 4). The length of the obtained *cox*1 sequences ranged from 777 to 801 bp (259 to 267 amino acids, respectively).

### Phylogenetic reconstruction

**Fig. 3.**
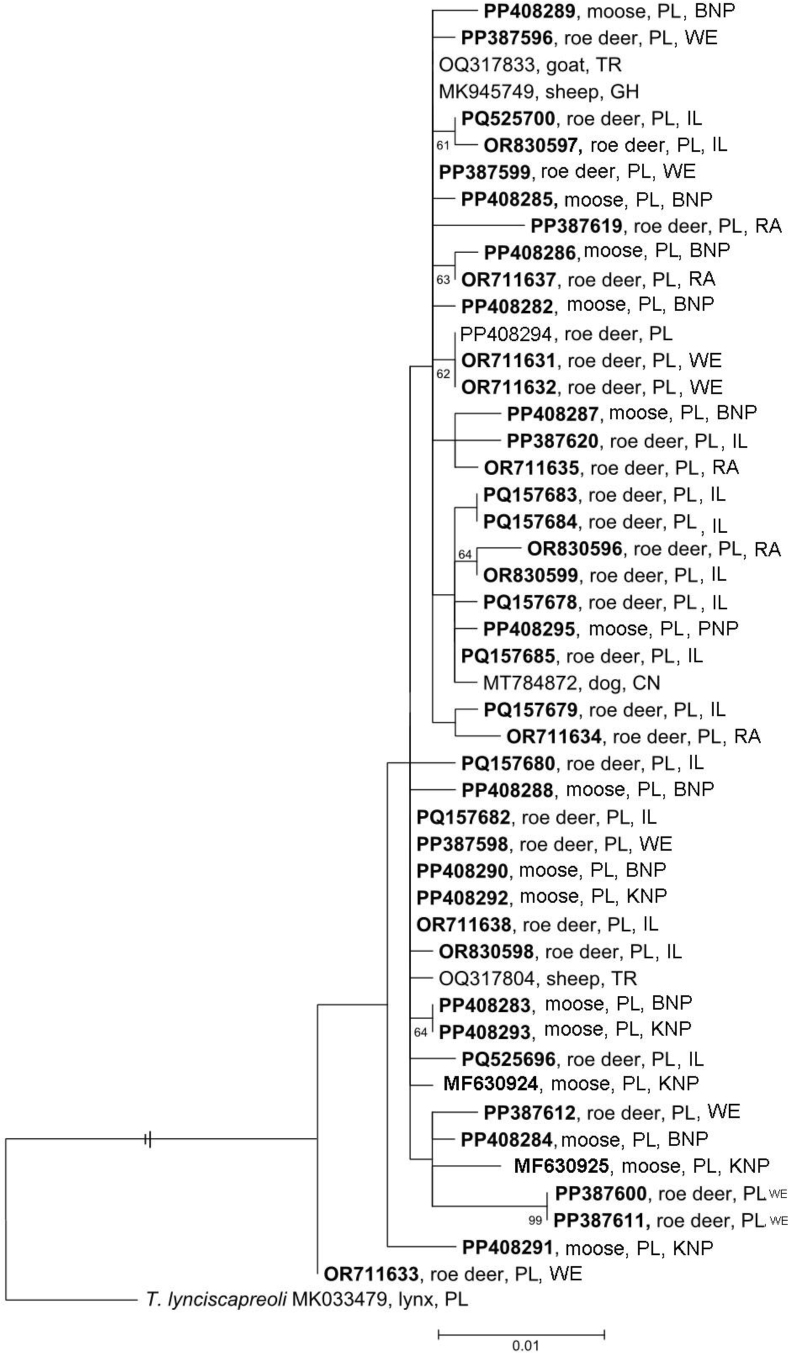
Maximum-likelihood phylogenetic analysis of the cytochrome *c* oxidase subunit 1 (*cox*1) data (776 bp) for *Taenia hydatigena* metacestodes found in roe deer and moose in Poland (model = General Time Reversible, GTR+I (invariant sites) +G (Γ distribution)). Branch support estimated using nonparametric bootstrap analyses based on 1,000 replicates. The outgroup was *Taenia lynciscapreoli*. Two-letter, six-number codes are GenBank accession numbers. Sequences obtained in this study are shown in bold. TR – originating in Türkiye; GH – originating in Ghana; PL – originating in Poland; CN – originating in China; BNP – Biebrza National Park; WE – Węgrów region; IL – Iłża region; RA – Rawa Mazowiecka region; PNP – Polesie National Park; KNP – Kampinos National Park

Maximum likelihood analysis of *cox*1 sequence data of *T. hydatigena*, with *T. lynciscapreoli* as an outgroup, revealed the isolates of *T. hydatigena* to cluster in three clades (Fig. 3). Subclades were noted within clades. The sequence of *T. hydatigena* isolated from roe deer inhabiting the Węgrów region in Poland (GenBank accession No. OR711633) diverged early. Genetic diversity was noted between isolates of *T. hydatigena* derived from the same animal. Specifically, it was revealed most dramatically in the metacestodes isolated from moose inhabiting Biebrza National Park, as their sequences were grouped in different clades and subclades (GenBank accession Nos PP408282–PP408290). Diversity to a lesser extent was shown in roe deer isolates of unitary origin: in a few cases, sequences of *T. hydatigena* from the same geographical location and animal were together in one subgroup (*i.e*. roe deer from the Węgrów region, GenBank Nos PP387600, PP387611, OR711631 and OR711632). Generally, the sequences from different animals and geographical locations were grouped in various clades and subclades, without any significant pattern.

## Discussion

Both *T. hydatigena* and *T. lynciscapreoli* create morphologically indistinguishable bladder-like cysts in the visceral organs of their intermediate host, and the similarity precludes identification by gross examination; therefore, species identification should be determined molecularly and/or morphologically after detailed measurements of the rostellar hooks of the larva (6, 24). In this study, exclusively *T. hydatigena* metacestodes were diagnosed in all infected roe deer and moose. This result may indirectly indicate the absence of the Eurasian lynx, the typical definitive host for *T. lynciscapreoli* (10), and perhaps the presence of the wolf, one of the typical definitive hosts for *T. hydatigena* in the sylvatic environment (34), in the locations where the study was conducted. In recent years, wolves have been observed in the vicinity of the roe deer and moose sample collection sites (26). In contrast, lynxes were observed only in the vicinity of some of the sites sites where moose samples were collected, *i.e*. in the national parks (28). Both species of carnivores are strictly protected in Poland. Thus, the suggestion can be made that the species composition of metacestodes in game species may indicate the occurrence of those protected carnivores in the monitored area.

In this study, mitochondrial *cox*1 sequences of various isolates revealed intraspecific genetic diversity in *T. hydatigena*, with the divergence reaching 1.42%. The result is consistent with previous data from Slovakia and Poland reporting the highest genetic divergence within the *cox*1 gene between isolates of *T. hydatigena* to be 1.3% and 1.5%, respectively (6, 10). Phylogenetic analysis of *T. hydatigena* isolates revealed that neither the host nor the geographical region determined isolate clustering within the clades. The observation was made that some isolates from animals inhabiting different regions were in the same subgroup, perhaps explained by contamination of extensive regions with the parasite’s eggs by the same individual of the definitive host species as it migrates. In other cases, isolates derived from the same individual created separate subclades, supporting the assumption that independent infections of *T. hydatigena* from various sources can accumulate in the same ungulate.

The study revealed that the omentum was the most prevalent location of metacestodes in roe deer, and that the mesentery was the counterpart in moose. These locations of *T. hydatigena* cysticerci were predominant in sheep in Egypt (1), but metacestodes were found most often in the livers of sheep, chamois, roe deer, fallow deer and wild boar in Slovakia (11) and wild boar in Italy (35), and here and in the mediastinum of moose in Poland (6) and red deer in Turkey (3).

The prevalence of infection noted in the current study ranged from 6.7% to 12.2% depending on the geographic location and host. The result was within the range observed in previous reports from Poland (11.1%) (6) and Italy (6.8%) (35). In contrast, a higher 21% prevalence was noted in sheep in Egypt (1), and a lower one of 3.9% in pigs in Africa (25). According to the authors of both surveys, access to potential definitive hosts impacts the prevalence in intermediate hosts. The higher prevalence of *T. hydatigena* in sheep in Egypt may result from the rearing of sheep there in small flocks, protected by one or two dogs. Egyptian *Taenia hydatigena* has ready access to definitive hosts in those dogs. Regarding the lower prevalence found in Africa, the genetic variability of *T. hydatigena* isolates between continents may result in differential adaptation to a dog-pig lifecycle, as evidenced by the far higher prevalence in both pigs and dogs in Asia (25). Therefore, it can be assumed that definitive host tropism is partly determined by *T. hydatigena* genotype. Infection with *Taenia hydatigena* larvae in roe deer and moose is mostly asymptomatic; however, if clinical signs appear, their severity depends on the number of larvae infecting the animal (3). Intensive infection may cause liver damage, peritonitis or emaciation as a result of hepatic dysfunction (33). Furthermore, the presence of larvae in some cervid organs, such as the lungs, may increase the probability of the animal being hunted by wolves, as infected individuals can exhibit reduced fitness and altered behaviour (19). In cases of heavy infestation, the meat of infected wild cervids may be considered unfit for human consumption and can negatively affect the economic value of hunting, despite *T. hydatigena* itself not posing a direct risk to human health (28).

In being based on over 200 animals, this to the best of our knowledge is the first large-scale study on *T. hydatigena* infection in wild ruminants in Poland. Further monitoring of *Taenia* spp. in their sylvatic cycles is important from both an epidemiological and public health perspective.

## Conclusion

The investigated roe deer and moose were infected with *T. hydatigena* isolates with a certain degree of genetic variability. In all cases, the infection prevalence was rather low, oscillating around 10%. So was the infection intensity, ranging from one to nine cysts. Although *T. hydatigena* is not a zoonotic agent, and the carcasses of infected game can be declared fit for human consumption after excision of lesions, its spread should be monitored and limited. Dogs should not be fed raw game meat in order to prevent parasitosis from spreading to farm animals. Also, roe deer acquisition of and infestation by *T. hydatigena* should be the subject of more detailed research, as the number of infected roe deer could be significant. During the 2022–2023 hunting season, 192,000 European roe deer were hunted (29). Were prevalence across all of Poland to be equal to that observed in our study (9.6%), the number of infected roe deer in Poland could exceed 18,000.

## Supplementary Material

Supplementary Material Details
