# Supplementary material for: Cysticercus tenuicollis in selected locations in Poland: genetic diversity, prevalence and epidemiological patterns in roe deer (Capreolus capreolus) and moose (Alces alces)
Source: J Vet Res. 2026 Feb 12;70(1):81–9. doi: 10.2478/jvetres-2026-0007 (PMC13054750; doi:10.2478/jvetres-2026-0007)
Supplement: Supplementary file 1 — Supplementary Material Details [file jvetres-2026-0007_sm.pdf]

**Supplementary Table 1.** Similarity % of the analysed nucleotide sequences of *Taenia hydatigena* (776 bp) and the *Taenia lynciscapreoli* sequence (MK033479) used in the phylogenetic tree as an outgroup, according to GenBank accession numbers. The BLASTR NCBI program (https://blast.ncbi.nlm.nih.gov/Blast.cgi) was used for comparison

| | Strain | 1 PQ157678 | 2 PQ157679 | 3 PQ157680 | 4 PQ157682 | 5 PQ157683 | 6 PQ157684 | 7 PQ157685 | 8 PQ525696 | 9 PQ525700 | 10 PP387600 | 11 PP387611 | 12 PP387612 | 13 PP408284 | 14 MF630925 | 15 PP387598 | 16 PP408288 | 17 PP408290 | 18 PP408292 | 19 OR711634 | 20 OR711638 | 21 OR830598 | 22 OQ317804 | 23 MF630924 | 24 PP408283 | 25 PP408293 | 26 PP387599 | 27 PP387619 | 28 PP387620 | 29 PP408287 | 30 OR711635 | 31 PP408282 | 32 PP408285 | 33 PP408286 | 34 OR711637 | 35 PP408289 | 36 PP408294 | 37 OR711631 | 38 OR711632 | 39 PP408295 | 40 MT784872 | 41 OR830596 | 42 OR830599 | 43 PP387596 | 44 MK945749 | 45 OQ317833 | 46 OR830597 | 47 PP408291 | 48 OR711633 | 49 MK033479 |
|---|---|---|---|---|---|---|---|---|---|---|---|---|---|---|---|---|---|---|---|---|---|---|---|---|---|---|---|---|---|---|---|---|---|---|---|---|---|---|---|---|---|---|---|---|---|---|---|---|---|---|
| 1 | PQ157678 | 100.00 | 99.48 | 99.36 | 99.61 | 99.74 | 99.74 | 99.87 | 99.36 | 99.61 | 98.84 | 98.84 | 99.23 | 99.36 | 99.10 | 99.61 | 99.36 | 99.61 | 99.61 | 99.36 | 99.61 | 99.48 | 99.48 | 99.48 | 99.48 | 99.48 | 99.74 | 99.23 | 99.36 | 99.36 | 99.48 | 99.61 | 99.61 | 99.48 | 99.61 | 99.48 | 99.61 | 99.61 | 99.61 | 99.74 | 99.74 | 99.48 | 99.74 | 99.61 | 99.74 | 99.74 | 99.48 | 99.10 | 99.10 | 93.56 |
| 2 | PQ157679 | 99.48 | 100.00 | 99.36 | 99.61 | 99.48 | 99.48 | 99.61 | 99.36 | 99.61 | 98.84 | 98.84 | 99.23 | 99.36 | 99.10 | 99.61 | 99.36 | 99.61 | 99.61 | 99.61 | 99.61 | 99.48 | 99.48 | 99.48 | 99.48 | 99.48 | 99.74 | 99.23 | 99.36 | 99.36 | 99.48 | 99.61 | 99.61 | 99.48 | 99.61 | 99.48 | 99.61 | 99.61 | 99.61 | 99.48 | 99.48 | 99.23 | 99.48 | 99.61 | 99.74 | 99.74 | 99.48 | 99.10 | 99.10 | 93.81 |
| 3 | PQ157680 | 99.36 | 99.36 | 100.00 | 99.74 | 99.61 | 99.61 | 99.48 | 99.48 | 99.48 | 98.97 | 98.97 | 99.36 | 99.48 | 99.23 | 99.74 | 99.48 | 99.74 | 99.74 | 99.48 | 99.74 | 99.61 | 99.61 | 99.61 | 99.61 | 99.61 | 99.61 | 99.10 | 99.23 | 99.23 | 99.36 | 99.48 | 99.48 | 99.36 | 99.48 | 99.36 | 99.48 | 99.48 | 99.48 | 99.36 | 99.36 | 99.10 | 99.36 | 99.48 | 99.61 | 99.61 | 99.36 | 99.23 | 99.23 | 93.69 |
| 4 | PQ157682 | 99.61 | 99.61 | 99.74 | 100.00 | 99.87 | 99.87 | 99.74 | 99.74 | 99.74 | 99.36 | 99.36 | 99.61 | 99.74 | 99.48 | 100.00 | 99.74 | 100.00 | 100.00 | 99.74 | 100.00 | 99.87 | 99.87 | 99.87 | 99.87 | 99.87 | 99.87 | 99.36 | 99.48 | 99.48 | 99.61 | 99.74 | 99.74 | 99.61 | 99.74 | 99.61 | 99.74 | 99.74 | 99.74 | 99.61 | 99.61 | 99.36 | 99.61 | 99.74 | 99.87 | 99.87 | 99.61 | 99.48 | 99.48 | 93.94 |
| 5 | PQ157683 | 99.74 | 99.48 | 99.61 | 99.87 | 100.00 | 100.00 | 99.87 | 99.61 | 99.61 | 99.10 | 99.10 | 99.48 | 99.61 | 99.36 | 99.87 | 99.61 | 99.87 | 99.87 | 99.61 | 99.87 | 99.74 | 99.74 | 99.74 | 99.74 | 99.74 | 99.74 | 99.23 | 99.36 | 99.36 | 99.48 | 99.61 | 99.61 | 99.48 | 99.61 | 99.48 | 99.61 | 99.61 | 99.61 | 99.74 | 99.74 | 99.48 | 99.74 | 99.61 | 99.74 | 99.74 | 99.48 | 99.36 | 99.36 | 93.81 |
| 6 | PQ157684 | 99.74 | 99.48 | 99.61 | 99.87 | 100.00 | 100.00 | 99.87 | 99.61 | 99.61 | 99.10 | 99.10 | 99.48 | 99.61 | 99.36 | 99.87 | 99.61 | 99.87 | 99.87 | 99.61 | 99.87 | 99.74 | 99.74 | 99.74 | 99.74 | 99.74 | 99.74 | 99.23 | 99.36 | 99.36 | 99.48 | 99.61 | 99.61 | 99.48 | 99.61 | 99.48 | 99.61 | 99.61 | 99.61 | 99.74 | 99.74 | 99.48 | 99.74 | 99.61 | 99.74 | 99.74 | 99.48 | 99.36 | 99.36 | 93.81 |
| 7 | PQ157685 | 99.87 | 99.61 | 99.48 | 99.74 | 99.87 | 99.87 | 100.00 | 99.48 | 99.74 | 98.97 | 98.97 | 99.36 | 99.48 | 99.23 | 99.74 | 99.48 | 99.74 | 99.74 | 99.48 | 99.74 | 99.61 | 99.61 | 99.61 | 99.61 | 99.61 | 99.87 | 99.36 | 99.48 | 99.48 | 99.61 | 99.74 | 99.74 | 99.61 | 99.74 | 99.61 | 99.74 | 99.74 | 99.74 | 99.87 | 99.87 | 99.61 | 99.87 | 99.74 | 99.87 | 99.87 | 99.61 | 99.23 | 99.23 | 93.69 |
| 8 | PQ525696 | 99.36 | 99.36 | 99.48 | 99.74 | 99.61 | 99.61 | 99.48 | 100.00 | 99.48 | 98.97 | 98.97 | 99.36 | 99.48 | 99.23 | 99.74 | 99.48 | 99.74 | 99.74 | 99.48 | 99.74 | 99.61 | 99.61 | 99.61 | 99.61 | 99.61 | 99.61 | 99.10 | 99.23 | 99.23 | 99.36 | 99.48 | 99.48 | 99.36 | 99.48 | 99.36 | 99.48 | 99.48 | 99.48 | 99.36 | 99.36 | 99.23 | 99.36 | 99.48 | 99.61 | 99.61 | 99.36 | 99.23 | 99.23 | 93.94 |
| 9 | PQ525700 | 99.61 | 99.61 | 99.48 | 99.74 | 99.61 | 99.61 | 99.74 | 99.48 | 100.00 | 98.97 | 98.97 | 99.36 | 99.48 | 99.23 | 99.74 | 99.48 | 99.74 | 99.74 | 99.48 | 99.74 | 99.61 | 99.61 | 99.61 | 99.61 | 99.61 | 99.87 | 99.36 | 99.48 | 99.48 | 99.61 | 99.74 | 99.74 | 99.61 | 99.74 | 99.61 | 99.74 | 99.74 | 99.74 | 99.61 | 99.61 | 99.36 | 99.61 | 99.74 | 99.87 | 99.87 | 99.87 | 99.23 | 99.23 | 93.69 |
| 10 | PP387600 | 98.84 | 98.84 | 98.97 | 99.23 | 99.10 | 99.10 | 98.97 | 98.97 | 98.97 | 100.00 | 100.00 | 99.10 | 99.23 | 99.10 | 99.23 | 98.97 | 99.23 | 99.23 | 98.97 | 99.23 | 99.10 | 99.10 | 99.23 | 99.10 | 99.10 | 99.10 | 98.58 | 98.71 | 98.71 | 98.84 | 98.97 | 98.97 | 98.84 | 98.97 | 98.84 | 99.23 | 99.23 | 99.23 | 98.84 | 98.84 | 98.58 | 98.84 | 98.97 | 99.10 | 99.10 | 98.84 | 98.97 | 98.71 | 93.81 |
| 11 | PP387611 | 98.84 | 98.84 | 98.97 | 99.23 | 99.10 | 99.10 | 98.97 | 98.97 | 98.97 | 100.00 | 100.00 | 99.10 | 99.23 | 99.10 | 99.23 | 98.97 | 99.23 | 99.23 | 98.97 | 99.23 | 99.10 | 99.10 | 99.23 | 99.10 | 99.10 | 99.10 | 98.58 | 98.71 | 98.71 | 98.84 | 98.97 | 98.97 | 98.84 | 98.97 | 98.84 | 99.23 | 99.23 | 99.23 | 98.84 | 98.84 | 98.58 | 98.84 | 98.97 | 99.10 | 99.10 | 98.84 | 98.97 | 98.71 | 93.81 |
| 12 | PP387612 | 99.23 | 99.23 | 99.36 | 99.61 | 99.48 | 99.48 | 99.36 | 99.36 | 99.36 | 99.10 | 99.10 | 100.00 | 99.61 | 99.36 | 99.61 | 99.36 | 99.61 | 99.61 | 99.36 | 99.61 | 99.48 | 99.48 | 99.48 | 99.48 | 99.48 | 99.48 | 98.97 | 99.10 | 99.10 | 99.23 | 99.36 | 99.36 | 99.23 | 99.36 | 99.23 | 99.36 | 99.36 | 99.36 | 99.23 | 99.23 | 98.97 | 99.23 | 99.36 | 99.48 | 99.48 | 99.23 | 99.10 | 99.10 | 93.56 |
| 13 | PP408284 | 99.36 | 99.36 | 99.48 | 99.74 | 99.61 | 99.61 | 99.48 | 99.48 | 99.48 | 99.23 | 99.23 | 99.61 | 100.00 | 99.48 | 99.74 | 99.48 | 99.74 | 99.74 | 99.48 | 99.74 | 99.61 | 99.61 | 99.61 | 99.61 | 99.61 | 99.61 | 99.10 | 99.23 | 99.23 | 99.36 | 99.48 | 99.48 | 99.36 | 99.48 | 99.36 | 99.48 | 99.48 | 99.48 | 99.36 | 99.36 | 99.10 | 99.36 | 99.48 | 99.61 | 99.61 | 99.36 | 99.23 | 99.23 | 93.81 |
| 14 | MF630925 | 99.10 | 99.10 | 99.23 | 99.48 | 99.36 | 99.36 | 99.23 | 99.23 | 99.23 | 99.10 | 99.10 | 99.36 | 99.48 | 100.00 | 99.48 | 99.23 | 99.48 | 99.48 | 99.23 | 99.48 | 99.36 | 99.36 | 99.61 | 99.36 | 99.36 | 99.36 | 98.84 | 98.97 | 98.97 | 99.10 | 99.36 | 99.23 | 99.10 | 99.23 | 99.10 | 99.23 | 99.23 | 99.23 | 99.10 | 99.10 | 98.84 | 99.10 | 99.23 | 99.36 | 99.36 | 99.10 | 98.97 | 99.23 | 94.07 |
| 15 | PP387598 | 99.61 | 99.61 | 99.74 | 100.00 | 99.87 | 99.87 | 99.74 | 99.74 | 99.74 | 99.23 | 99.23 | 99.61 | 99.74 | 99.48 | 100.00 | 99.74 | 100.00 | 100.00 | 99.74 | 100.00 | 99.87 | 99.87 | 99.87 | 99.87 | 99.87 | 99.87 | 99.36 | 99.48 | 99.48 | 99.61 | 99.74 | 99.74 | 99.61 | 99.74 | 99.61 | 99.74 | 99.74 | 99.74 | 99.61 | 99.61 | 99.36 | 99.61 | 99.74 | 99.87 | 99.87 | 99.61 | 99.48 | 99.48 | 93.94 |
| 16 | PP408288 | 99.36 | 99.36 | 99.48 | 99.74 | 99.61 | 99.61 | 99.48 | 99.48 | 99.48 | 98.97 | 98.97 | 99.36 | 99.48 | 99.23 | 99.74 | 100.00 | 99.74 | 99.74 | 99.48 | 99.74 | 99.61 | 99.61 | 99.61 | 99.61 | 99.61 | 99.61 | 99.10 | 99.23 | 99.23 | 99.36 | 99.48 | 99.48 | 99.36 | 99.48 | 99.36 | 99.48 | 99.48 | 99.48 | 99.36 | 99.36 | 99.10 | 99.36 | 99.48 | 99.61 | 99.61 | 99.36 | 99.23 | 99.23 | 93.69 |
| 17 | PP408290 | 99.61 | 99.61 | 99.74 | 100.00 | 99.87 | 99.87 | 99.74 | 99.74 | 99.74 | 99.23 | 99.23 | 99.61 | 99.74 | 99.48 | 100.00 | 99.74 | 100.00 | 100.00 | 99.74 | 100.00 | 99.87 | 99.87 | 99.87 | 99.87 | 99.87 | 99.87 | 99.36 | 99.48 | 99.48 | 99.61 | 99.74 | 99.74 | 99.61 | 99.74 | 99.61 | 99.74 | 99.74 | 99.74 | 99.61 | 99.61 | 99.36 | 99.61 | 99.74 | 99.87 | 99.87 | 99.61 | 99.48 | 99.48 | 93.94 |
| 18 | PP408292 | 99.61 | 99.61 | 99.74 | 100.00 | 99.87 | 99.87 | 99.74 | 99.74 | 99.74 | 99.23 | 99.23 | 99.61 | 99.74 | 99.48 | 100.00 | 99.74 | 100.00 | 100.00 | 99.74 | 100.00 | 99.87 | 99.87 | 99.87 | 99.87 | 99.87 | 99.87 | 99.36 | 99.48 | 99.48 | 99.61 | 99.74 | 99.74 | 99.61 | 99.74 | 99.61 | 99.74 | 99.74 | 99.74 | 99.61 | 99.61 | 99.36 | 99.61 | 99.74 | 99.87 | 99.87 | 99.61 | 99.48 | 99.48 | 93.94 |
| 19 | OR711634 | 99.36 | 99.61 | 99.48 | 99.74 | 99.61 | 99.61 | 99.48 | 99.48 | 99.48 | 98.97 | 98.97 | 99.36 | 99.48 | 99.23 | 99.74 | 99.48 | 99.74 | 99.74 | 100.00 | 99.74 | 99.61 | 99.61 | 99.61 | 99.61 | 99.61 | 99.61 | 99.10 | 99.23 | 99.23 | 99.36 | 99.48 | 99.48 | 99.36 | 99.48 | 99.36 | 99.48 | 99.48 | 99.48 | 99.36 | 99.36 | 99.10 | 99.36 | 99.48 | 99.61 | 99.61 | 99.36 | 99.23 | 99.23 | 94.20 |
| 20 | OR711638 | 99.61 | 99.61 | 99.74 | 100.00 | 99.87 | 99.87 | 99.74 | 99.74 | 99.74 | 99.23 | 99.23 | 99.61 | 99.74 | 99.48 | 100.00 | 99.74 | 100.00 | 100.00 | 99.74 | 100.00 | 99.87 | 99.87 | 99.87 | 99.87 | 99.87 | 99.87 | 99.36 | 99.48 | 99.48 | 99.61 | 99.74 | 99.74 | 99.61 | 99.74 | 99.61 | 99.74 | 99.74 | 99.74 | 99.61 | 99.61 | 99.36 | 99.61 | 99.74 | 99.87 | 99.87 | 99.61 | 99.48 | 99.48 | 93.94 |
| 21 | OR830598 | 99.48 | 99.48 | 99.61 | 99.87 | 99.74 | 99.74 | 99.61 | 99.61 | 99.61 | 99.10 | 99.10 | 99.48 | 99.61 | 99.36 | 99.87 | 99.61 | 99.87 | 99.87 | 99.61 | 99.87 | 100.00 | 99.74 | 99.74 | 99.74 | 99.74 | 99.74 | 99.23 | 99.36 | 99.36 | 99.48 | 99.61 | 99.61 | 99.48 | 99.61 | 99.48 | 99.61 | 99.61 | 99.61 | 99.48 | 99.48 | 99.23 | 99.48 | 99.61 | 99.74 | 99.74 | 99.48 | 99.36 | 99.36 | 93.81 |
| 22 | OQ317804 | 99.48 | 99.48 | 99.61 | 99.87 | 99.74 | 99.74 | 99.61 | 99.61 | 99.61 | 99.10 | 99.10 | 99.48 | 99.61 | 99.36 | 99.87 | 99.61 | 99.87 | 99.87 | 99.61 | 99.87 | 99.74 | 100.00 | 99.74 | 99.74 | 99.74 | 99.74 | 99.23 | 99.36 | 99.36 | 99.48 | 99.61 | 99.61 | 99.48 | 99.61 | 99.48 | 99.61 | 99.61 | 99.61 | 99.48 | 99.48 | 99.23 | 99.48 | 99.61 | 99.74 | 99.74 | 99.48 | 99.36 | 99.36 | 93.81 |
| 23 | MF630924 | 99.48 | 99.48 | 99.61 | 99.87 | 99.74 | 99.74 | 99.61 | 99.61 | 99.61 | 99.23 | 99.23 | 99.48 | 99.61 | 99.61 | 99.87 | 99.61 | 99.87 | 99.87 | 99.61 | 99.87 | 99.74 | 99.74 | 100.00 | 99.74 | 99.74 | 99.74 | 99.23 | 99.36 | 99.36 | 99.48 | 99.61 | 99.61 | 99.48 | 99.61 | 99.48 | 99.61 | 99.61 | 99.61 | 99.48 | 99.48 | 99.23 | 99.48 | 99.61 | 99.74 | 99.74 | 99.48 | 99.36 | 99.36 | 94.07 |
| 24 | PP408283 | 99.48 | 99.48 | 99.61 | 99.87 | 99.74 | 99.74 | 99.61 | 99.61 | 99.61 | 99.10 | 99.10 | 99.48 | 99.61 | 99.36 | 99.87 | 99.61 | 99.87 | 99.87 | 99.61 | 99.87 | 99.74 | 99.74 | 99.74 | 100.00 | 100.00 | 99.74 | 99.23 | 99.36 | 99.36 | 99.48 | 99.61 | 99.61 | 99.48 | 99.61 | 99.48 | 99.61 | 99.61 | 99.61 | 99.48 | 99.48 | 99.23 | 99.48 | 99.61 | 99.74 | 99.74 | 99.48 | 99.36 | 99.36 | 93.81 |
| 25 | PP408293 | 99.48 | 99.48 | 99.61 | 99.87 | 99.74 | 99.74 | 99.61 | 99.61 | 99.61 | 99.10 | 99.10 | 99.48 | 99.61 | 99.36 | 99.87 | 99.61 | 99.87 | 99.87 | 99.61 | 99.87 | 99.74 | 99.74 | 99.74 | 100.00 | 100.00 | 99.74 | 99.23 | 99.36 | 99.36 | 99.48 | 99.61 | 99.61 | 99.48 | 99.61 | 99.48 | 99.61 | 99.61 | 99.61 | 99.48 | 99.48 | 99.23 | 99.48 | 99.61 | 99.74 | 99.74 | 99.48 | 99.36 | 99.36 | 93.81 |
| 26 | PP387599 | 99.74 | 99.74 | 99.61 | 99.87 | 99.74 | 99.74 | 99.87 | 99.61 | 99.87 | 99.10 | 99.10 | 99.48 | 99.61 | 99.36 | 99.87 | 99.61 | 99.87 | 99.87 | 99.61 | 99.87 | 99.74 | 99.74 | 99.74 | 99.74 | 99.74 | 100.00 | 99.48 | 99.61 | 99.61 | 99.74 | 99.87 | 99.87 | 99.74 | 99.87 | 99.74 | 99.87 | 99.87 | 99.87 | 99.74 | 99.74 | 99.48 | 99.74 | 99.87 | 100.00 | 100.00 | 99.74 | 99.36 | 99.36 | 93.81 |
| 27 | PP387619 | 99.23 | 99.23 | 99.10 | 99.36 | 99.23 | 99.23 | 99.36 | 99.10 | 99.36 | 98.58 | 98.58 | 98.97 | 99.10 | 98.84 | 99.36 | 99.10 | 99.36 | 99.36 | 99.10 | 99.36 | 99.23 | 99.23 | 99.23 | 99.23 | 99.23 | 99.48 | 100.00 | 99.10 | 99.10 | 99.23 | 99.36 | 99.36 | 99.23 | 99.36 | 99.23 | 99.36 | 99.36 | 99.36 | 99.23 | 99.23 | 98.97 | 99.23 | 99.36 | 99.48 | 99.48 | 99.23 | 98.84 | 98.84 | 93.56 |
| 28 | PP387620 | 99.36 | 99.36 | 99.23 | 99.48 | 99.36 | 99.36 | 99.48 | 99.23 | 99.48 | 98.71 | 98.71 | 99.10 | 99.23 | 98.97 | 99.48 | 99.23 | 99.48 | 99.48 | 99.23 | 99.48 | 99.36 | 99.36 | 99.36 | 99.36 | 99.36 | 99.61 | 99.10 | 100.00 | 99.48 | 99.61 | 99.48 | 99.48 | 99.36 | 99.48 | 99.36 | 99.48 | 99.48 | 99.48 | 99.36 | 99.36 | 99.10 | 99.36 | 99.48 | 99.61 | 99.61 | 99.36 | 98.97 | 98.97 | 93.94 |
| 29 | PP408287 | 99.36 | 99.36 | 99.23 | 99.48 | 99.36 | 99.36 | 99.48 | 99.23 | 99.48 | 98.71 | 98.71 | 99.10 | 99.23 | 98.97 | 99.48 | 99.23 | 99.48 | 99.48 | 99.23 | 99.48 | 99.36 | 99.36 | 99.36 | 99.36 | 99.36 | 99.61 | 99.10 | 99.48 | 100.00 | 99.61 | 99.48 | 99.48 | 99.36 | 99.48 | 99.36 | 99.48 | 99.48 | 99.48 | 99.36 | 99.36 | 99.10 | 99.36 | 99.48 | 99.61 | 99.61 | 99.36 | 98.97 | 99.23 | 93.94 |
| 30 | OR711635 | 99.48 | 99.48 | 99.36 | 99.61 | 99.48 | 99.48 | 99.61 | 99.36 | 99.61 | 98.84 | 98.84 | 99.23 | 99.36 | 99.10 | 99.61 | 99.36 | 99.61 | 99.61 | 99.36 | 99.61 | 99.48 | 99.48 | 99.48 | 99.48 | 99.48 | 99.74 | 99.23 | 99.61 | 99.61 | 100.00 | 99.61 | 99.61 | 99.48 | 99.61 | 99.48 | 99.61 | 99.61 | 99.61 | 99.48 | 99.48 | 99.23 | 99.48 | 99.61 | 99.74 | 99.74 | 99.48 | 99.10 | 99.10 | 94.07 |
| 31 | PP408282 | 99.61 | 99.61 | 99.48 | 99.74 | 99.61 | 99.61 | 99.74 | 99.48 | 99.74 | 98.97 | 98.97 | 99.36 | 99.48 | 99.36 | 99.74 | 99.48 | 99.74 | 99.74 | 99.48 | 99.74 | 99.61 | 99.61 | 99.61 | 99.61 | 99.61 | 99.87 | 99.36 | 99.48 | 99.48 | 99.61 | 100.00 | 99.74 | 99.61 | 99.74 | 99.61 | 99.74 | 99.74 | 99.74 | 99.61 | 99.61 | 99.36 | 99.61 | 99.74 | 99.87 | 99.87 | 99.61 | 99.23 | 99.23 | 93.94 |
| 32 | PP408285 | 99.61 | 99.61 | 99.48 | 99.74 | 99.61 | 99.61 | 99.74 | 99.48 | 99.74 | 98.97 | 98.97 | 99.36 | 99.48 | 99.23 | 99.74 | 99.48 | 99.74 | 99.74 | 99.48 | 99.74 | 99.61 | 99.61 | 99.61 | 99.61 | 99.61 | 99.87 | 99.36 | 99.48 | 99.48 | 99.61 | 99.74 | 100.00 | 99.61 | 99.74 | 99.61 | 99.74 | 99.74 | 99.74 | 99.61 | 99.61 | 99.36 | 99.61 | 99.74 | 99.87 | 99.87 | 99.61 | 99.23 | 99.23 | 93.69 |
| 33 | PP408286 | 99.48 | 99.48 | 99.36 | 99.61 | 99.48 | 99.48 | 99.61 | 99.36 | 99.61 | 98.84 | 98.84 | 99.23 | 99.36 | 99.10 | 99.61 | 99.36 | 99.61 | 99.61 | 99.36 | 99.61 | 99.48 | 99.48 | 99.48 | 99.48 | 99.48 | 99.74 | 99.23 | 99.36 | 99.36 | 99.48 | 99.61 | 99.61 | 100.00 | 99.87 | 99.48 | 99.61 | 99.61 | 99.61 | 99.48 | 99.48 | 99.23 | 99.48 | 99.61 | 99.74 | 99.74 | 99.48 | 99.10 | 99.10 | 93.81 |
| 34 | OR711637 | 99.61 | 99.61 | 99.48 | 99.74 | 99.61 | 99.61 | 99.74 | 99.48 | 99.74 | 98.97 | 98.97 | 99.36 | 99.48 | 99.23 | 99.74 | 99.48 | 99.74 | 99.74 | 99.48 | 99.74 | 99.61 | 99.61 | 99.61 | 99.61 | 99.61 | 99.87 | 99.36 | 99.48 | 99.48 | 99.61 | 99.74 | 99.74 | 99.87 | 100.00 | 99.61 | 99.74 | 99.74 | 99.74 | 99.61 | 99.61 | 99.36 | 99.61 | 99.74 | 99.87 | 99.87 | 99.61 | 99.23 | 99.23 | 93.94 |
| 35 | PP408289 | 99.48 | 99.48 | 99.36 | 99.61 | 99.48 | 99.48 | 99.61 | 99.61 | 99.36 | 98.84 | 98.84 | 99.23 | 99.36 | 99.10 | 99.61 | 99.36 | 99.61 | 99.61 | 99.36 | 99.61 | 99.48 | 99.48 | 99.48 | 99.48 | 99.48 | 99.74 | 99.23 | 99.36 | 99.36 | 99.48 | 99.61 | 99.61 | 99.48 | 99.61 | 100.00 | 99.61 | 99.61 | 99.61 | 99.48 | 99.48 | 99.23 | 99.48 | 99.61 | 99.74 | 99.74 | 99.48 | 99.10 | 99.10 | 93.56 |
| 36 | PP408294 | 99.61 | 99.61 | 99.48 | 99.74 | 99.61 | 99.61 | 99.74 | 99.48 | 99.74 | 99.23 | 99.23 | 99.36 | 99.48 | 99.23 | 99.74 | 99.48 | 99.74 | 99.74 | 99.48 | 99.74 | 99.61 | 99.61 | 99.61 | 99.61 | 99.61 | 99.87 | 99.36 | 99.48 | 99.48 | 99.61 | 99.74 | 99.74 | 99.61 | 99.74 | 99.61 | 100.00 | 100.00 | 100.00 | 99.61 | 99.61 | 99.36 | 99.61 | 99.74 | 99.87 | 99.87 | 99.61 | 99.23 | 99.23 | 93.94 |
| 37 | OR711631 | 99.61 | 99.61 | 99.48 | 99.74 | 99.61 | 99.61 | 99.74 | 99.48 | 99.74 | 99.23 | 99.23 | 99.36 | 99.48 | 99.23 | 99.74 | 99.48 | 99.74 | 99.74 | 99.48 | 99.74 | 99.61 | 99.61 | 99.61 | 99.61 | 99.61 | 99.87 | 99.36 | 99.48 | 99.48 | 99.61 | 99.74 | 99.74 | 99.61 | 99.74 | 99.61 | 100.00 | 100.00 | 100.00 | 99.61 | 99.61 | 99.36 | 99.61 | 99.74 | 99.87 | 99.87 | 99.61 | 99.23 | 99.23 | 93.94 |
| 38 | OR711632 | 99.61 | 99.61 | 99.48 | 99.74 | 99.61 | 99.61 | 99.74 | 99.48 | 99.74 | 99.23 | 99.23 | 99.36 | 99.48 | 99.23 | 99.74 | 99.48 | 99.74 | 99.74 | 99.48 | 99.74 | 99.61 | 99.61 | 99.61 | 99.61 | 99.61 | 99.87 | 99.36 | 99.48 | 99.48 | 99.61 | 99.74 | 99.74 | 99.61 | 99.74 | 99.61 | 100.00 | 100.00 | 100.00 | 99.61 | 99.61 | 99.36 | 99.61 | 99.74 | 99.87 | 99.87 | 99.61 | 99.23 | 99.23 | 93.94 |
| 39 | PP408295 | 99.74 | 99.48 | 99.36 | 99.61 | 99.74 | 99.74 | 99.87 | 99.36 | 99.61 | 98.84 | 98.84 | 99.23 | 99.36 | 99.10 | 99.61 | 99.36 | 99.61 | 99.61 | 99.36 | 99.61 | 99.48 | 99.48 | 99.48 | 99.48 | 99.48 | 99.74 | 99.23 | 99.36 | 99.36 | 99.48 | 99.61 | 99.61 | 99.48 | 99.61 | 99.48 | 99.61 | 99.61 | 99.61 | 100.00 | 99.74 | 99.48 | 99.74 | 99.61 | 99.74 | 99.74 | 99.48 | 99.10 | 99.10 | 93.56 |
| 40 | MT784872 | 99.74 | 99.48 | 99.61 | 99.36 | 99.74 | 99.74 | 99.87 | 99.36 | 99.61 | 98.84 | 98.84 | 99.23 | 99.36 | 99.10 | 99.61 | 99.36 | 99.61 | 99.61 | 99.36 | 99.61 | 99.48 | 99.48 | 99.48 | 99.48 | 99.48 | 99.74 | 99.23 | 99.36 | 99.36 | 99.48 | 99.61 | 99.61 | 99.48 | 99.61 | 99.48 | 99.61 | 99.61 | 99.61 | 99.74 | 100.00 | 99.48 | 99.74 | 99.61 | 99.74 | 99.74 | 99.48 | 99.10 | 99.10 | 93.56 |
| 41 | OR830596 | 99.48 | 99.23 | 99.10 | 99.36 | 99.48 | 99.48 | 99.61 | 99.23 | 99.36 | 98.58 | 98.58 | 98.97 | 99.10 | 98.84 | 99.36 | 99.10 | 99.36 | 99.36 | 99.10 | 99.36 | 99.23 | 99.23 | 99.23 | 99.23 | 99.23 | 99.48 | 98.97 | 99.10 | 99.10 | 99.23 | 99.36 | 99.36 | 99.23 | 99.36 | 99.23 | 99.36 | 99.36 | 99.36 | 99.48 | 99.48 | 100.00 | 99.74 | 99.36 | 99.48 | 99.48 | 99.23 | 98.84 | 98.84 | 93.30 |
| 42 | OR830599 | 99.74 | 99.48 | 99.36 | 99.61 | 99.74 | 99.74 | 99.87 | 99.36 | 99.61 | 98.84 | 98.84 | 99.23 | 99.36 | 99.10 | 99.61 | 99.36 | 99.61 | 99.61 | 99.36 | 99.61 | 99.48 | 99.48 | 99.48 | 99.48 | 99.48 | 99.74 | 99.23 | 99.36 | 99.36 | 99.48 | 99.61 | 99.61 | 99.48 | 99.61 | 99.48 | 99.61 | 99.61 | 99.61 | 99.74 | 99.74 | 99.74 | 100.00 | 99.61 | 99.74 | 99.74 | 99.48 | 99.10 | 99.10 | 93.56 |
| 43 | PP387596 | 99.61 | 99.61 | 99.48 | 99.74 | 99.61 | 99.61 | 99.74 | 99.48 | 99.74 | 98.97 | 98.97 | 99.36 | 99.48 | 99.23 | 99.74 | 99.48 | 99.74 | 99.74 | 99.48 | 99.74 | 99.61 | 99.61 | 99.61 | 99.61 | 99.61 | 99.87 | 99.36 | 99.48 | 99.48 | 99.61 | 99.74 | 99.74 | 99.61 | 99.74 | 99.61 | 99.74 | 99.74 | 99.74 | 99.61 | 99.61 | 99.36 | 99.61 | 100.00 | 99.87 | 99.87 | 99.61 | 99.23 | 99.23 | 93.69 |
| 44 | MK945749 | 99.74 | 99.74 | 99.61 | 99.87 | 99.74 | 99.74 | 99.87 | 99.61 | 99.87 | 99.10 | 99.10 | 99.48 | 99.61 | 99.36 | 99.87 | 99.61 | 99.87 | 99.87 | 99.61 | 99.87 | 99.74 | 99.74 | 99.74 | 99.74 | 99.74 | 100.00 | 99.48 | 99.61 | 99.61 | 99.74 | 99.87 | 99.87 | 99.74 | 99.87 | 99.74 | 99.87 | 99.87 | 99.87 | 99.74 | 99.74 | 99.48 | 99.74 | 99.87 | 100.00 | 100.00 | 99.74 | 99.36 | 99.36 | 93.81 |
| 45 | OQ317833 | 99.74 | 99.74 | 99.61 | 99.87 | 99.74 | 99.74 | 99.87 | 99.61 | 99.87 | 99.10 | 99.10 | 99.48 | 99.61 | 99.36 | 99.87 | 99.61 | 99.87 | 99.87 | 99.61 | 99.87 | 99.74 | 99.74 | 99.74 | 99.74 | 99.74 | 100.00 | 99.48 | 99.61 | 99.61 | 99.74 | 99.87 | 99.87 | 99.74 | 99.87 | 99.74 | 99.87 | 99.87 | 99.87 | 99.74 | 99.74 | 99.48 | 99.74 | 99.87 | 100.00 | 100.00 | 99.74 | 99.36 | 99.36 | 93.81 |
| 46 | OR830597 | 99.48 | 99.48 | 99.36 | 99.61 | 99.48 | 99.48 | 99.61 | 99.36 | 99.87 | 98.84 | 98.84 | 99.23 | 99.36 | 99.10 | 99.61 | 99.36 | 99.61 | 99.61 | 99.36 | 99.61 | 99.48 | 99.48 | 99.48 | 99.48 | 99.48 | 99.74 | 99.23 | 99.36 | 99.36 | 99.48 | 99.61 | 99.61 | 99.48 | 99.61 | 99.48 | 99.61 | 99.61 | 99.61 | 99.48 | 99.48 | 99.23 | 99.48 | 99.61 | 99.74 | 99.74 | 100.00 | 99.10 | 99.10 | 93.56 |
| 47 | PP408291 | 99.10 | 99.10 | 99.23 | 99.48 | 99.36 | 99.36 | 99.23 | 99.23 | 99.23 | 98.97 | 98.97 | 99.10 | 99.23 | 98.97 | 99.48 | 99.23 | 99.48 | 99.48 | 99.23 | 99.48 | 99.36 | 99.36 | 99.36 | 99.36 | 99.36 | 99.36 | 98.84 | 98.97 | 98.97 | 99.10 | 99.23 | 99.23 | 99.10 | 99.23 | 99.10 | 99.23 | 99.23 | 99.23 | 99.10 | 99.10 | 98.84 | 99.10 | 99.23 | 99.36 | 99.36 | 99.10 | 100.00 | 99.23 | 94.07 |
| 48 | OR711633 | 99.10 | 99.10 | 99.23 | 99.48 | 99.36 | 99.36 | 99.23 | 99.23 | 99.23 | 98.71 | 98.71 | 99.10 | 99.23 | 99.23 | 99.48 | 99.23 | 99.48 | 99.48 | 99.23 | 99.48 | 99.36 | 99.36 | 99.36 | 99.36 | 99.36 | 99.36 | 98.84 | 98.97 | 99.23 | 99.10 | 99.23 | 99.23 | 99.10 | 99.23 | 99.10 | 99.23 | 99.23 | 99.23 | 99.10 | 99.10 | 98.84 | 99.10 | 99.23 | 99.36 | 99.36 | 99.10 | 99.23 | 100.00 | 94.20 |
| 49 | MK033479 | 93.56 | 93.81 | 93.69 | 93.94 | 93.81 | 93.81 | 93.69 | 93.94 | 93.69 | 93.81 | 93.81 | 93.56 | 93.81 | 94.07 | 93.94 | 93.69 | 93.94 | 93.94 | 94.20 | 93.94 | 93.81 | 93.81 | 94.07 | 93.81 | 93.81 | 93.81 | 93.56 | 93.94 | 93.94 | 94.07 | 93.94 | 93.69 | 93.81 | 93.94 | 93.56 | 93.94 | 93.94 | 93.94 | 93.56 | 93.56 | 93.30 | 93.56 | 93.69 | 93.81 | 93.81 | 93.56 | 94.07 | 94.20 | 100.00 |
| | Strain | 1 PQ157678 | 2 PQ157679 | 3 PQ157680 | 4 PQ157682 | 5 PQ157683 | 6 PQ157684 | 7 PQ157685 | 8 PQ525696 | 9 PQ525700 | 10 PP387600 | 11 PP387611 | 12 PP387612 | 13 PP408284 | 14 MF630925 | 15 PP387598 | 16 PP408288 | 17 PP408290 | 18 PP408292 | 19 OR711634 | 20 OR711638 | 21 OR830598 | 22 OQ317804 | 23 MF630924 | 24 PP408283 | 25 PP408293 | 26 PP387599 | 27 PP387619 | 28 PP387620 | 29 PP408287 | 30 OR711635 | 31 PP408282 | 32 PP408285 | 33 PP408286 | 34 OR711637 | 35 PP408289 | 36 PP408294 | 37 OR711631 | 38 OR711632 | 39 PP408295 | 40 MT784872 | 41 OR830596 | 42 OR830599 | 43 PP387596 | 44 MK945749 | 45 OQ317833 | 46 OR830597 | 47 PP408291 | 48 OR711633 | 49 MK033479 |
